# Local adherent technique for transplanting mesenchymal stem cells as a potential treatment of cartilage defect

**DOI:** 10.1186/ar2460

**Published:** 2008-07-29

**Authors:** Hideyuki Koga, Masayuki Shimaya, Takeshi Muneta, Akimoto Nimura, Toshiyuki Morito, Masaya Hayashi, Shiro Suzuki, Young-Jin Ju, Tomoyuki Mochizuki, Ichiro Sekiya

**Affiliations:** 1 Section of Orthopedic Surgery, Graduate School, Tokyo Medical and Dental University, 1-5-45 Yushima, Bunkyo-ku, Tokyo 113-8519, Japan; 2 Global Center of Excellence Program, International Research Center for Molecular Science in Tooth and Bone Diseases, Tokyo Medical and Dental University, 1-5-45 Yushima, Bunkyo-ku, Tokyo 113-8519, Japan; 3 Section of Cartilage Regeneration, Graduate School, Tokyo Medical and Dental University, 1-5-45 Yushima, Bunkyo-ku, Tokyo 113-8519, Japan

## Abstract

**Introduction:**

Current cell therapy for cartilage regeneration requires invasive procedures, periosteal coverage and scaffold use. We have developed a novel transplantation method with synovial mesenchymal stem cells (MSCs) to adhere to the cartilage defect.

**Methods:**

For *ex vivo *analysis in rabbits, the cartilage defect was faced upward, filled with synovial MSC suspension, and held stationary for 2.5 to 15 minutes. The number of attached cells was examined. For *in vivo *analysis in rabbits, an autologous synovial MSC suspension was placed on the cartilage defect, and the position was maintained for 10 minutes to adhere the cells to the defect. For the control, either the same cell suspension was injected intra-articularly or the defects were left empty. The three groups were compared macroscopically and histologically. For *ex vivo *analysis in humans, in addition to the similar experiment in rabbits, the expression and effects of neutralizing antibodies for adhesion molecules were examined.

**Results:**

*Ex vivo *analysis in rabbits demonstrated that the number of attached cells increased in a time-dependent manner, and more than 60% of cells attached within 10 minutes. The *in vivo *study showed that a large number of transplanted synovial MSCs attached to the defect at 1 day, and the cartilage defect improved at 24 weeks. The histological score was consistently better than the scores of the two control groups (same cell suspension injected intra-articularly or defects left empty) at 4, 12, and 24 weeks. *Ex vivo *analysis in humans provided similar results to those in rabbits. Intercellular adhesion molecule 1-positive cells increased between 1 minute and 10 minutes, and neutralizing antibodies for intercellular adhesion molecule 1, vascular cell adhesion molecule 1 and activated leukocyte-cell adhesion molecule inhibited the attachment.

**Conclusion:**

Placing MSC suspension on the cartilage defect for 10 minutes resulted in adherence of >60% of synovial MSCs to the defect, and promoted cartilage regeneration. This adherent method makes it possible to adhere MSCs with low invasion, without periosteal coverage, and without a scaffold.

## Introduction

Various methods have been reported for the treatment of articular cartilage injury. Marrow stimulation techniques [1,2] are the most prevalent, but defects are often filled with fibrous cartilage and the repaired cartilage later degenerates [3]. Autologous osteochondral transplantation [4] and chondrocyte transplantation [5] can regenerate hyaline cartilage; however, the invasiveness of the procedures is of concern [6,7], thereby limiting such applications for the repair of large defects.

Mesenchymal stem cells (MSCs) are an attractive cell source for cartilage regenerative medicine because they can be harvested in a minimally invasive manner, are easily isolated and expanded, and have multipotentiality that includes chondrogenesis [8-10]. In addition, synovial MSCs are especially promising due to their high proliferative capacity and chondrogenic potential [11-16].

Treatment with chondrocytes and MSCs requires the transplantation of a cell and scaffold composite with a periosteum covering, and is presently a common repair method [17,18]. The method is extremely invasive, however, with a long incision to the skin and capsule to harvest the periosteum, transplantation of the cell/gel composite, and fixation with suturing to the neighboring cartilage. With periosteal coverage, hypertrophy and ossification are of concern [17]. The most popular scaffold is currently composed of collagen gel, which is produced by type I collagen derived from animal skins, thereby introducing the risk of disease transmission and immune reaction [19].

We developed a novel transplantation procedure with synovial MSCs for cartilage regeneration. The degree of surgical invasion is as minimal as the marrow stimulation techniques, since our procedure can also be performed arthroscopically. Scaffolds are not necessary, thereby increasing the safety and economic feasibility. Our study will advance and extend the clinical application of MSC-based cell therapy for cartilage injury.

## Materials and methods

### Rabbits

Skeletally mature Japanese White Rabbits weighing approximately 3.2 kg (ranging from 2.8 to 3.6 kg) were used in the experiments. Animal care was in accordance with the guidelines of the animal committee of Tokyo Medical and Dental University. The operation was performed under anesthesia induced by intramuscular injection of 25 mg/kg ketamine hydrochloride and intravenous injection of 45 mg/kg sodium pentobarbital.

### Isolation and culture of synovial mesenchymal stem cells in rabbits

Synovium with the subsynovial tissue was harvested from the left knee of the rabbits under anesthesia. The synovium was digested in a 3 mg/ml collagenase D solution (Roche Diagnostics, Mannheim, Germany) in αMEM (Invitrogen Corp., Carlsbad, CA, USA) at 37°C. After 3 hours, digested cells were filtered through a 70-μm nylon filter (Becton Dickinson, Franklin Lakes, NJ, USA), and the remaining tissues were discarded. The digested cells were plated at 5 × 10^4 ^cells/cm^2 ^in 150 cm^2 ^culture dishes (Nalge Nunc International, Rochester, NY, USA) in complete culture medium, αMEM containing 10% FBS (lot selected for rapid growth of bone marrow derived MSCs, 100 units/ml penicillin, 100 μg/ml streptomycin, and 250 ng/ml amphotericin B; Invitrogen Corp.), and were incubated at 37°C with 5% humidified CO_2_. After 3 to 4 days, the medium was changed to remove nonadherent cells, and the adherent cells were cultured for 7 days as passage 0 without refeeding. The cells were then trypsinized, harvested and resuspended to be used for transplantation. We already reported that these cells had characteristics of MSCs [20-22].

The cells that were transplanted in animals to be sacrificed at day 1 were labeled for cell tracking by the fluorescent lipophilic tracer 1,1'-dioctadecyl-3,3,3',3'-tetramethylindocarbocyanine perchlorate (DiI) (Molecular Probes, Eugene, OR, USA). For labeling, the cells were resuspended at 1 × 10^6 ^cells/ml in αMEM, and DiI was added at 5 μl/ml in αMEM. After incubation for 20 minutes at 37°C with 5% humidified CO_2_, the cells were centrifuged at 450 × *g *for 5 min and washed twice with PBS [20,23], and the cells were then resuspended in PBS for the transplantation.

### *Ex vivo *sequential analysis of the number of attached cells in rabbits

Full-thickness osteochondral defects (5 mm × 5 mm wide, 3 mm deep) were created in the trochlear groove of the femurs of adult rabbits. The distal end of the femurs were then removed, and were precultured in serum-free Dulbecco's MEM (Invitrogen) supplemented with 100 units/ml penicillin (Invitrogen), 100 μg/ml streptomycin (Invitrogen), and 250 ng/ml amphotericin B (Invitrogen) for 24 hours. To determine the length of time needed for cell attachment to the defect, the cartilage defect of the femoral condyle was faced upward. Passage 0 autologous synovial MSCs, precultured for 7 days, were used for the transplantation.

**Figure 1:**
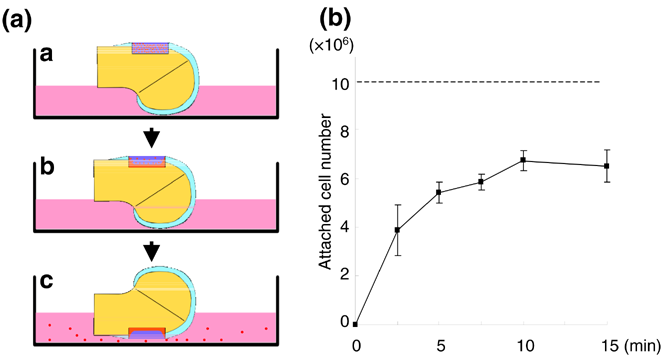
*Ex vivo *sequential analysis of cell attachment to rabbit cartilage defects by local adherent technique. **(a) **Scheme for the method: image a, cartilage defect of the femoral condyle was faced upward and the defect was filled with 10^6 ^1,1'-dioctadecyl-3,3,3',3'-tetramethylindocarbocyanine perchlorate-labeled rabbit synovial mesenchymal stem cells in 100 μl PBS; image b, defect was held stationary for 2.5, 5, 7.5, 10, and 15 minutes; image c, femur was turned with the defect side down for 10 minutes so that nonadhered cells in the defect fell in the culture medium. The nonattached cell number was then determined, and the attached cell number was extrapolated. **(b) **Cell number attached to the cartilage defects by the local adherent technique. Data expressed as the mean ± standard deviation (*n* = 3).

The defect was filled with DiI-labeled synovial MSC suspension, which consisted of 10^7 ^cells in 100 μl PBS, and was left stationary for 2.5, 5, 7.5, 10, and 15 minutes. The femurs were then turned with the defect side down for 10 minutes. This allowed the nonadhered cells in the defect to discard the defect in the culture medium (Figure 1a). The nonadhered cells in the medium were collected, as were the nonadhered cells attached to the dishes after trypsinization. The total number of nonadhered cells positive for DiI was counted. Finally, the adhered cell number attached to the cartilage defects was calculated by subtracting from 10 × 10^6 ^cells.

### *In vivo *transplantation

Thirty-six rabbits were used for the *in vivo *transplantation study. Autologous synovial MSC transplantation was performed 7 days after the harvest. Under anesthesia, the right knee joint was approached through a medial parapatellar incision, and the patella was dislocated laterally. Full-thickness osteochondral defects (5 mm × 5 mm wide, 3 mm deep), whose size were critical for rabbit knees [24], were created in the trochlear groove of the femur.

The animals were divided into three groups for transplantation. For the control group, the cartilage defect was left empty. For the intra-articular group, 10^7 ^DiI-labeled autologous synovial MSCs in 100 μl PBS were injected into the knee joint after the capsule was closed. For the local adherent group, the defect was filled with the cell suspension of 10^7^DiI-labeled autologous synovial MSCs in 100 μl PBS and held stationary for 10 minutes with the defect upward. In no groups were the defects patched, and a periosteum or artificial membrane was not used. All rabbits were returned to their cages after the operation and were allowed to move freely. Animals were sacrificed with an overdose of sodium pentobarbital at 1 day and 4, 12, and 24 weeks after the operation (*n* = 3 at each time point).

### Macroscopic examination

The cartilage defects were examined macroscopically for color, integrity and smoothness. Osteoarthritic changes and synovitis of the knee were also investigated. Macroscopic pictures of the femoral condyles were taken for evaluation using MPS-7 (Sugiura Laboratory Inc., Tokyo, Japan), a dedicated medical photography platform. Digital images were taken using a Nikon Coolpix 4500 digital camera (Nikon, Tokyo, Japan).

### Histological examination and fluorescent microscopic examination

The dissected distal femurs were immediately fixed in a 4% paraformaldehyde solution. The specimens were decalcified in 4% ethylenediamine tetraacetic acid solution, dehydrated with a gradient ethanol series, and embedded in paraffin blocks. Sagittal sections 5 μm thick were obtained from the center of each defect and were stained with toluidine blue. Sections dedicated for fluorescent microscopic visualization of DiI-labeled cells were not stained with toluidine blue, and nuclei were counterstained by 4',6-diamidino-2-phenylindole dihydrochloride.

### Histological score

Histological sections of the repaired tissue were analyzed using a grading system consisting of five categories (cell morphology, matrix staining, surface regularity, cartilage thickness, and integration of donor with host), which were modified from the repaired cartilage score described by Wakitani and colleagues [25], so that overly thick regenerated cartilage could not be overestimated (Table 1). The scoring was performed in a blinded manner by two observers, and there was no significant interobserver difference.

**Table 1:** Histological scoring system for cartilage repair

Category	Points
Cell morphology	
Hyaline cartilage	4
Mostly hyaline cartilage	3
Mostly fibrocartilage	2
Mostly non-cartilage	1
Noncartilage only	0
Matrix-staining (metachromasia)	
Normal (compared with host adjacent cartilage)	3
Slightly reduces	2
Markedly reduced	1
No metachromatic stain	0
Surface regularity^a^	
Smooth (>3/4)	3
Moderate (1/2 to 3/4)	2
Irregular (1/4 to 1/2)	1
Severely irregular (<1/4)	0
Thickness of cartilage^b^	
2/3 to 4/3	3
5/3 to 4/3	2
1/3 to 2/3 or >5/3	1
<1/3	0
Integration of donor with host adjacent cartilage	
Both edges integrated	2
One edge integrated	1
Neither edge integrated	0
Total maximum	15

^a^Total smooth area of the reparative cartilage compared with the entire area of the cartilage defect. ^b^Average thickness of the reparative cartilage compared with that of the surrounding cartilage.

### *Ex vivo *sequential analysis of the number of attached cells in humans

The study was approved by our Institutional Review Board, and informed consent was obtained from all study subjects. Human synovium and cartilage were harvested during total knee arthroplasty with medial compartment osteoarthritis. Synovial tissue was minced into small pieces, digested in a collagenase solution, and then filtered. Nucleated cells were cultured for 14 days. Passage 3 cells were used for further analyses [15].

Osteochondral fragments at the lateral femoral condyle were diced with a bone saw. The cartilage defects 2.5 mm in diameter were created and filled with 800 × 10^3 ^DiI-labeled human synovial MSCs in 8 μl PBS. After 5, 10, 20, and 30 minutes, the cartilage defects were turned down for 10 minutes. After trypsinization, the DiI-positive cells in the dish were counted, and number of the cell attached to the cartilage defects was calculated by subtracting from 800 × 10^3 ^cells.

### Immunohistochemistry

The sections of the human osteochondral fragments were deparaffinized, washed in PBS, and pretreated with 0.4 mg/ml proteinase K (DAKO, Carpinteria, CA, USA) in Tris–HCl buffer for 15 minutes at room temperature. Endogenous peroxidases were quenched using 3% hydrogen peroxide in methanol for 20 minutes at room temperature. The sections were rinsed three times in PBS for 5 minutes and were briefly blocked with 5% normal horse or rabbit serum (Vector Laboratories, Burlingame, CA, USA) to avoid nonspecific binding of the antibody. The sections were then incubated in mouse monoclonal anti-human intercellular adhesion molecule 1 (ICAM-1) antibody (1:50 dilution; SANBIO BV, Uden, Netherlands) or in goat anti-human vascular adhesion molecule 1 (VCAM-1) antibody (1:100 dilution; R&D Systems, Wiesbarden, Germany) at room temperature for 1 hour. After rinsing in PBS, the tissues were incubated with biotinylated horse anti-mouse or rabbit anti-goat IgG secondary antibody (Vector Laboratories) for 30 minutes at room temperature. After incubation for another 30 minutes with Vectastain ABC reagent (Vector Laboratories), the slides were counterstained with Mayer hematoxylin, dehydrated, and mounted in a xylol-soluble mount (Vitro-Club; Langenbrinck, Emmendingen, Germany).

### Neutralizing antibodies for adhesion molecules in human samples

Three million DiI-labeled human synovial MSCs were incubated in 2 ml PBS including 1% BSA with 10 μg/ml neutralizing antibody for human ICAM-1, VCAM-1, activated leukocyte-cell adhesion molecule, or mouse IgG1 isotype control antibody (R&D Systems) for 30 minutes at 37°C with 5% humidified CO_2 _[26]. After the supernatant was discarded, 800 × 10^3 ^cells resuspended in 8 μl PBS were placed on the cartilage defect of osteocartilage fragment and held stationary for 10 minutes. The cartilage defects were then turned down for 10 minutes.

### ICAM-1 expression in synovial mesenchymal stem cells after plating on slide grasses

Human synovial MSCs at 500 × 10^3 ^in 10 μl PBS were placed on eight-well chamber glass slides (BD Bioscience) and washed by PBS at 1 minute and 10 minutes, and were then fixed with 99.5% acetone for 15 minutes. The glass slides were stained with mouse monoclonal anti-human ICAM-1 antibody (1:10 dilution with PBS in 5% goat serum; R&D Systems) for 2 hours. After rinsing with PBS three times, the slides were stained with goat anti-mouse IgG secondary antibody labeled with Alexa fluor 568 (Invitrogen) for 1 hour. The nuclei were stained with Hoechst 33342 (Invitrogen). The number of ICAM-1-positive cells and nuclei was counted in three high-power fields.

### Statistical analysis

To assess differences, the Kruskal–Wallis test and the Mann–Whitney U test were used. *P *< 0.05 was considered significant.

## Results

### *Ex vivo *analysis of the number of cells attached to cartilage defects in rabbits

To clarify the minimum time for an adequate number of synovial MSCs to attach to the cartilage defect by the local adherent technique, we performed an *ex vivo *sequential analysis using rabbit synovial MSCs and rabbit cartilage (Figure 1a). The number of attached cells increased in a time-dependent manner, and more than 60% of the cells attached in 10 minutes (Figure 1b).

### Macroscopic observation for the *in vivo *study

Osteochondral defects were created in rabbit knees. For the control group, the cartilage defect was left empty. For the intra-articular group, synovial MSCs were injected into the knee joint after the capsule was closed. For the local adherent group, the defect was filled with the synovial MSC suspension and faced upward for 10 minutes according to the *ex vivo *analyses.

At 1 day, the cartilage defects were overlaid with blood clots, and there seemed to be no obvious differences among the control, intra-articular, and local adherent groups macroscopically (data not shown).

**Figure 2:**
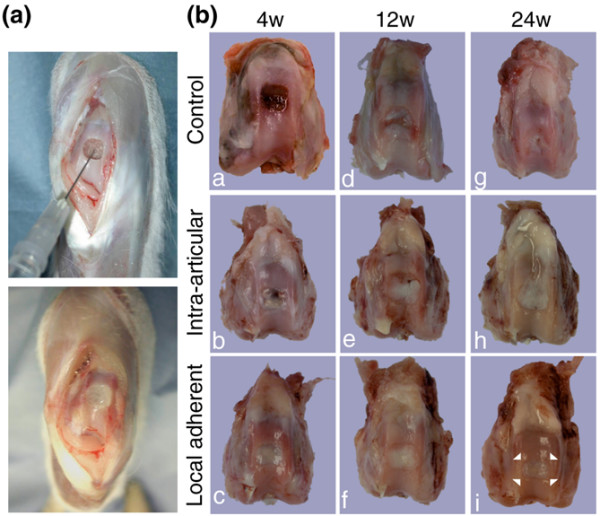
*In vivo *analysis of cartilage repair by synovial mesenchymal stem cell transplantation in rabbits. **(a) **Cell transplantation on a cartilage defect in a rabbit by the local adherent technique. The osteochondral defect was faced upward (upper panel), and the defect was filled with synovial mesenchymal stem cell (MSC) suspension (lower panel) and held stationary for 10 minutes for the cells to adhere. **(b) **Macroscopic observation of cartilage defects after cell transplantation. For the control group, the cartilage defect was left empty. For the intra-articular group, synovial MSCs were injected into the knee joint after the capsule was closed. For the local adherent group, the defect was filled with the synovial MSC suspension and held still for 10 minutes. Femoral condyles 4, 12 and 24 weeks post surgery are shown. The corners of the margin between repaired tissue and native cartilage are indicated as arrowheads in the local adherent group at 24 weeks.

At 4 weeks, the cartilage defect in the control group still showed reddish tissue (Figure 2b, image a). In the intra-articular group, the defect was covered with whitish tissue in some areas, but the reddish area remained in other areas (Figure 2b, image b). In the local adherent group, the defect became whitish and glossy in the entire area (Figure 2b, image c).

At 12 weeks, in the control and intra-articular groups, the reddish regions decreased in size but still remained locally (Figure 2b, images d and e). In the local adherent group, the border between repaired tissue and neighboring cartilage appeared less distinct (Figure 2b, image f).

At 24 weeks, the cartilage defect area in the control group decreased but still remained (Figure 2b, image g). In the intra-articular group, the defects were covered with whitish tissue but the margins were still distinct (Figure 2b, image h). In the local adherent group, the peripheral lesion of the defect appeared to integrate into the surrounding native cartilage (Figure 2b, image i).

In all three groups there were no obvious features of hydrarthrosis or synovial proliferation. Mild spur formation was observed on the edge of the trochlear groove of the femur in some samples of the control group, but there were no osteoarthritic changes of the femorotibial joint in any groups.

### Histological observation for *in vivo *study

**Figure 3:**
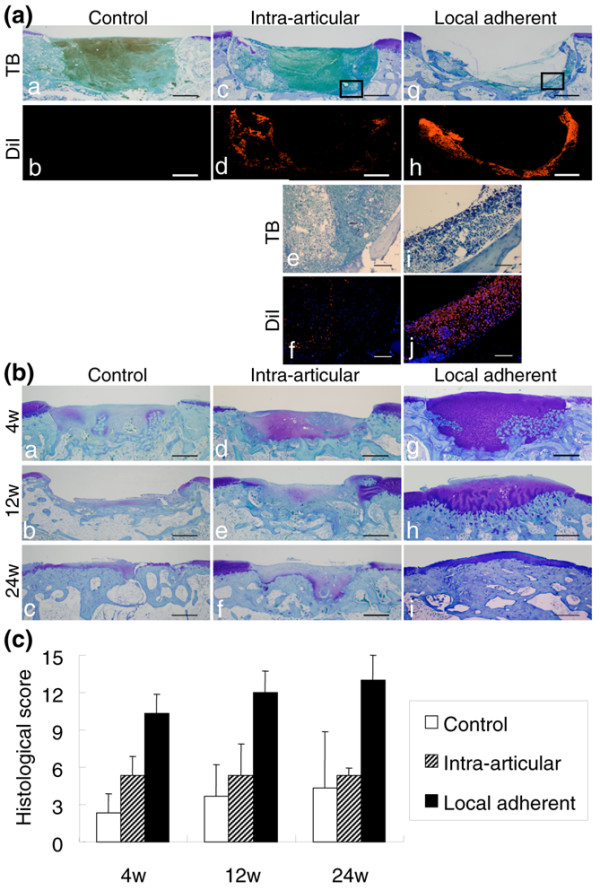
Histological analyses. **(a) **Observation 1 day after cell transplantation. Sagittal sections stained with Toluidine blue (TB) and the serial sections under fluorescence for the 1,1'-dioctadecyl-3,3,3',3'-tetramethylindocarbocyanine perchlorate (DiI) label are shown. Higher magnifications of the framed areas are shown in images e, f, i, and j. The nuclei were counterstained by 4',6-diamidino-2-phenylindole dihydrochloride in images f and j. Bars (a to d, g, h) = 1 mm; bars (e, f, i, j) = 100 μm. **(b) **Sagittal sections stained with TB. The distal side is shown on the right side of the image. Bars = 1 mm. **(c) **Histological score for the cartilage defect after cell transplantation. Histological findings were quantitated using the scoring system (Table 1), in which a full score was 15 and a higher score indicates cartilage repair. The scores of the local adherent group improved continuously through 24 weeks and were better than those of other groups at each point. Data expressed as the mean ± standard deviation (*n* = 3; *P *< 0.05 by Kruskal–Wallis test).

At 1 day, the defect in the control group was filled with blood clots (Figure 3a, images a and b). In the intra-articular group, DiI-positive synovial MSCs were observed in the defect (Figure 3a, images c and d); the cells were very sparse when examined at higher magnification, however, even in the selected area where relatively dense DiI-positive cells were observed in lower magnification (Figure 3a, images e and f). In contrast, in the local adherent group, there were more DiI-positive synovial MSCs along with the osteochondral defect, with the cellular layer 20 cells deep (Figure 3a, images g and h). DiI-positive cells were denser in the local adherent group (Figure 3a, images I and j) than in the intra-articular group.

At 4 weeks, the defect in the control group was filled with fibrous tissue and the cartilage matrix formation was poor (Figure 3b, image a). In the intra-articular group, although more cartilage matrix could be observed than in the control group, the height of the repaired tissue was lower than that of the surrounding cartilage (Figure 3b, image b). In the local adherent group, the defect was filled with abundant cartilage matrix. In addition in the local adherent group, remodeling of the cartilage into the underlying bone was observed in deep areas (Figure 3b, image c).

At 12 weeks, in the control and intra-articular groups, the defects were filled with fibrous tissues and were poorly healed (Figure 3b, images d and e). In the local adherent group, the cartilage matrix at the defect still remained, and the border between regenerated cartilage and subchondral bone moved upward. Integration between native cartilage and regenerated tissue appeared to be improved (Figure 3b, image f).

At 24 weeks, in the control and intra-articular groups, the cartilage defects were still not healed (Figure 3b, images g and h). In the local adherent group, the regenerated cartilage matrix was well developed. The subchondral bone moved further upward, and the thickness of the regenerated cartilage was similar to that of the neighboring cartilage. The borders between the native and regenerated tissue were well integrated (Figure 3b, image i).

The histological scores of the local adherent group improved continuously through 24 weeks and were always better than those of the control group and the intra-articular group at each point (Figure 3c).

### *Ex vivo *analysis of human synovial mesenchymal stem cell attachment to human cartilage defect

The results described above were obtained using rabbit MSCs. We investigated whether human MSC exhibited the same capacity as rabbit cells to adhere to cartilage with the same kinetics. The defects of cartilage obtained from humans were faced upward, filled with 800 × 10^3 ^DiI-labeled human synovial MSCs, and the position maintained for 5 to 30 minutes.

**Figure 4:**
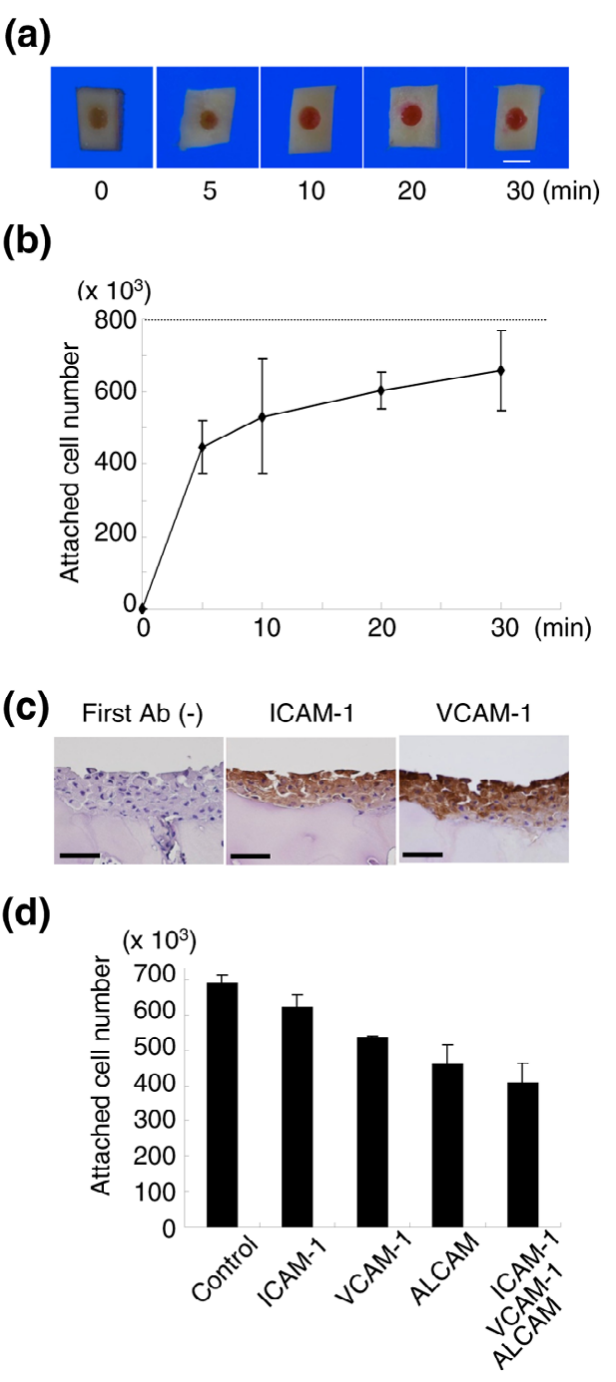
*Ex vivo *analysis of human synovial mesenchymal stem cell attachment to human cartilage defect. The cartilage defect at 2.5 mm diameter was faced upward, filled with 800 × 10^3 ^1,1'-dioctadecyl-3,3,3',3'-tetramethylindocarbocyanine perchlorate (DiI)-labeled human synovial mesenchymal stem cells (MSCs) in 8 μl PBS, and held stationary for 5, 10, 20, and 30 minutes. **(a) **Macroscopic features of cartilage defects filled with DiI-labeled human synovial MSCs for the indicated time. Bar = 2.5 mm. **(b) **Cell number attached to the cartilage defects. Data expressed as the mean ± standard deviation (*n* = 3). **(c) **Adhesion molecule expressions in cartilage defects filled with synovial MSC suspension for 10 minutes. Bars = 50 μm. Ab, antibody; ICAM-1, intercellular adhesion molecule 1; VCAM-1, vascular adhesion molecule 1. **(d) **Effects of neutralizing antibodies for adhesion molecules on attachment of human synovial MSCs on human cartilage defects. The cartilage defect was filled with DiI-labeled human synovial MSC suspension with control or neutralizing antibodies. After 10 minutes, the attached cell number was measured. Data expressed as the mean ± standard deviation (*n* = 3; *P *< 0.05 by Kruskal–Wallis test). ALCAM, activated leukocyte-cell adhesion molecule.

Macroscopically, the cartilage defect looked yellowish at time 0, slightly reddish at 5 minutes, and red at 10 minutes and thereafter (Figure 4a). The cell number attached to the cartilage defect increased rapidly at 5 minutes, and then started to rise slowly (Figure 4b). It should be noted that more than 60% of the human synovial MSCs already adhered to the cartilage defects at 10 minutes, indicating similarity between rabbits and humans.

### Adhesion molecules

**Figure 5:**
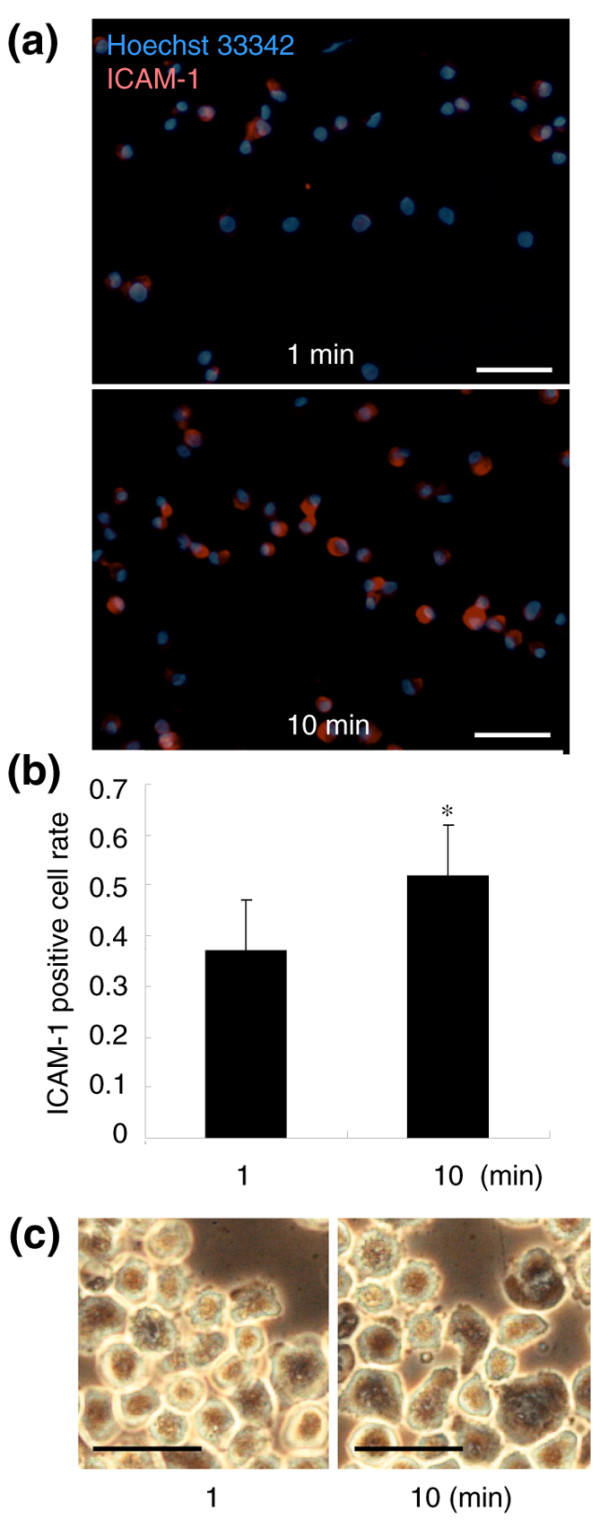
Molecular and morphological events during a 10-minute period. **(a) **Intercellular adhesion molecule 1 (ICAM-1) expression in human synovial mesenchymal stem cells (MSCs) 1 minute and 10 minutes after plating on glass slides. ICAM-1-positive cells are shown as light shading, and nuclei as dark shading. Bars = 100 μm. **(b) **ICAM-1-positive cell rate. The number of ICAM-1-positive cells and nuclei were counted in three high-power fields. Data expressed as the mean ± standard deviation (*P < 0.05 by Mann–Whitney U test). **(c) **Morphological alterations of human synovial MSCs between 1 minute and 10 minutes after plating on a culture dish. Bars = 50 μm.

It is expected that adhesion molecules are involved in cell attachment. Ten minutes after filling human synovial MSCs in human cartilage defect, adhered cells expressed ICAM-1 and VCAM-1 (Figure 4c). Neutralizing antibodies for ICAM-1, VCAM-1, and activated leukocyte-cell adhesion molecule, separately or together, inhibited attachment of human synovial MSCs to human cartilage defects (Figure 4d). When human synovial MSCs were plated on grass slides, ICAM-1-positive cells significantly increased between 1 minute and 10 minutes (Figure 5a,b).

### Morphological event during a 10 minute period

We finally examined the morphological change of human synovial MSCs during a 10-minute period after plating on a culture dish. Most cells looked thick and round at 1 minute. They became thinner, larger, and polygonal at 10 minutes (Figure 5c).

## Discussion

For successful cartilage regeneration with MSCs, a sufficient number of cells are required in the defect of the cartilage. The number of MSCs decreased along with the period during chondrogenesis *in vitro *[12,27] and *in vivo *[20] due to apoptosis of the MSCs [28]. Chondrogenic potential of MSCs depends on the cell number *in vitro *[29]. We previously reported that transplantation of synovial MSCs/gel composites with 5 × 10^7 ^cells/ml provided better results than transplantation of composites with 10^6 ^cells/ml for the similar cartilage defects in rabbits [21]. These findings indicate that transplanted MSCs do not increase, and a higher number of MSCs can provide better results for cartilage regeneration. In the present study we chose a dose of 10^8 ^cells/ml MSC suspension for the *ex vivo *and *in vivo *investigation. This concentration is the maximum for preparing cell suspension.

We previously created the same full-thickness cartilage defect in rabbits, and transplanted a synovial MSC/collagen gel complex, which was covered with periosteum. The defect was repaired successfully [20], and histological scores were similar using collagen gel and using the local adherence technique.

We believe that the local adherent technique is much less invasive and more attractive for clinical application.

Before we performed this research, we had speculated that intra-articular injection of MSCs might result in better improvement of the cartilage defect than it actually did. Practically, most of the intra-articular injected cells adhered to synovial tissue (data not shown), and only a small portion of the cells adhered to the cartilage defect. Injection of more cells would increase the number of cells that adhered to the defect; however, the injection of a large number of cells would also increase the number of cells that adhered to the synovium, thereby increasing the risk of adverse effects such as synovial proliferation. The local adherent technique we describe here made it possible to adhere the cells to the defect site more effectively than an intra-articular injection technique.

In this research, human synovial MSCs attached to the cartilage defect 10 minutes after plating already expressed ICAM-1 and VCAM-1, and neutralizing antibodies for ICAM-1, VCAM-1, or activated leukocyte-cell adhesion molecule inhibited the attachment. The ICAM-1-positive cell rate also increased 10 minutes after plating on glass slides. Attachment of synovial MSCs within 10 minutes was mediated by these adhesion molecules. Their modification may have increased the efficacy of cell attachment.

Our *ex vivo *studies demonstrated that more than 60% of synovial MSCs adhered to the cartilage defect after synovial MSC suspension was placed on the cartilage defect for 10 minutes both in humans and rabbits. The remaining nonadherent synovial MSCs seemed to attach to synovial tissue in the knee joint. When we injected 10^7 ^GFP-positive rat synovial MSCs into the knee with meniscal defect in rats, GFP-positive cells were observed in the meniscal defect and in the synovial tissues. GFP mRNA expressions were also detected in the synovium, but not in the brain, the lung, the liver, the kidney, and the spleen [30]. Furthermore, our *in vivo *imaging system could not be detected in any other organs expect the knee when luciferase-positive synovial MSCs were injected into normal rat knee (data not shown). These findings indicate that synovial MSCs transplanted into the knee are not distributed to other organs.

We previously compared the *in vivo *chondrogenic potential of synovial MSCs, bone marrow MSCs, adipose MSCs, and muscle MSCs by transplanting them into cartilage defects in rabbits. Synovial MSCs and bone marrow MSCs had much more chondrogenic potential than adipose MSCs and muscle MSCs [21]. For clinical safety, autologous human serum should be used instead of FBS. We recently reported that autologous human serum predominated in increasing the proliferation of human synovial MSCs rather than human bone marrow MSCs [16]. These results indicate that synovial MSCs and bone marrow MSCs are useful cell sources for cartilage regeneration, but it is easier to prepare a sufficient number in synovial MSCs than in bone marrow MSCs when autologous serum is used.

In the original autologous chondrocyte transplantation technique, the cartilage defect was covered with the periosteum and then chondrocyte suspension was injected into the defect [5]. One poor aspect of the autologous chondrocyte transplantation method was the leakage of the cell suspension; however, the original autologous chondrocyte transplantation method produced successful results. We speculate that chondrocytes in suspension might adhere to the cartilage defect soon after chondrocyte suspension is injected into the defect.

**Figure 6:**
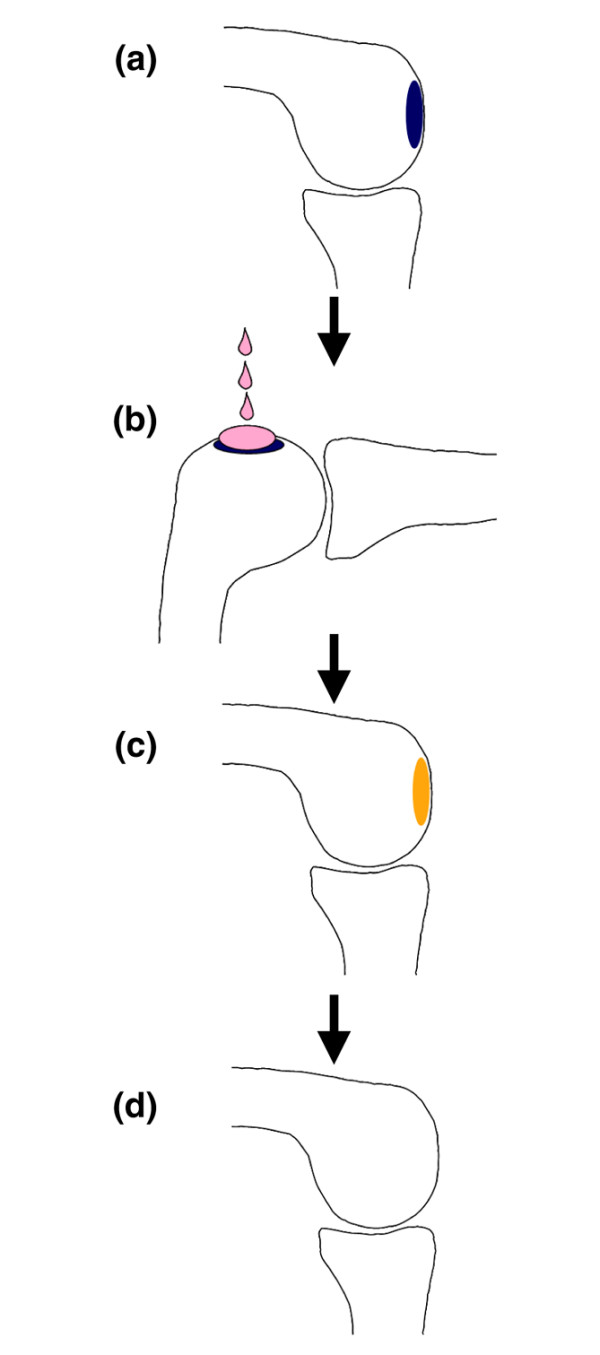
Application of low-invasive local adherent technique to transplant synovial mesenchymal stem cells into cartilage defect. **(a) **For illustration, the cartilage defect is located on the condyles of the femur in the knee joint. **(b) **Knee is positioned so that the cartilage defect is faced upward. The synovial mesenchymal stem cell (MSC) suspension is then slowly dripped onto the cartilage defect, and the knee is held stationary for 10 minutes. **(c) **Knee position is permitted to be changed, and the synovial MSCs have adhered to the cartilage defect. **(d) **Transplanted synovial MSCs differentiate according to the microenvironment, and the cartilage regenerates.

For clinical application, we summarize the local adhesion technique as follows. When the operation for the cartilage injury is performed (Figure 6a), the knee is positioned so that the cartilage defect is upward (Figure 6b). The synovial MSC suspension is then slowly dripped onto the cartilage defect and the knee is held stationary for 10 minutes. The knee position is then permitted to be changed and the synovial MSCs are adhered to the cartilage defect (Figure 6c). The transplanted synovial MSCs differentiate appropriately for the local microenvironment, and the cartilage regenerates (Figure 6d). Additionally, this procedure can be performed arthroscopically, without the need for additional scaffold, from the cell harvest to the transplantation. This protocol will advance and extend the clinical application of MSC-based cell therapy for cartilage injury.

## Conclusion

We developed a novel implantation procedure with synovial MSCs for cartilage regeneration. The local adherent technique could achieve successful cartilage regeneration with low invasion, without periosteal coverage, and without a scaffold. This will advance and extend clinical application of MSC-based cell therapy for cartilage injury.

## Abbreviations

BSA = bovine serum albumin; DiI = 1,1'-dioctadecyl-3,3,3',3'-tetramethylindocarbocyanine perchlorate; FBS = fetal bovine serum; GFP = green fluorescent protein; ICAM-1 = intercellular adhesion molecule 1; MEM, modified Eagle's medium; MSC = mesenchymal stem cell; PBS = phosphate-buffered saline; VCAM-1 = vascular cell adhesion molecule 1.

## Competing interests

The authors declare that they have no competing interests.

## Authors' contributions

HK and MS contributed equally to this work. HK carried out *ex vivo *and *in vivo *experiments in rabbits, analyzed the data, and drafted the manuscript. MS performed *ex vivo *experiments in humans and analyzed the data. TMu designed the initial plan. AN, TMor, MH, Y-JJ, and TMoc assisted in the animal experiments. SS assisted in the human experiments. IS conducted the experiments and completed the final manuscript. All authors read and approved the final manuscript.
